# Gonioscopy-assisted Transluminal Trabeculotomy (GATT) combined phacoemulsification surgery: Outcomes at a 2-year follow-up

**DOI:** 10.1038/s41433-022-02087-2

**Published:** 2022-05-24

**Authors:** Yue Wan, Kai Cao, Jin Wang, Yunxiao Sun, Rong Du, Ziyi Wang, Jidi Zhang, Huaizhou Wang, Ningli Wang

**Affiliations:** 1 Beijing Institute of Ophthalmology, Beijing Tongren Hospital, Capital Medical University, Beijing, China; 2 Beijing Tongren Eye Center, Beijing Tongren Hospital, Capital Medical University, Beijing, China

**Keywords:** Outcomes research, Optic nerve diseases

## Abstract

**Background/Objectives:**

This study aimed to provide a 24-month follow-up on the surgical success and safety of gonioscopy-assisted transluminal trabeculotomy (GATT) combined with phacoemulsification and intraocular lens (IOL) implantation in the treatment of patients with primary open-angle glaucoma (POAG) combined cataract.

**Subjects/Methods:**

We included 124 consecutive cases of POAG with microcatheter-assisted GATT or GATT combined with phacoemulsification and IOL implantation at Beijing Tongren Eye Centre between October 2019 and November 2020. Main outcome measures included surgical success rate, changes in IOP, number of antiglaucoma medications, best corrected visual acuity (BCVA), postoperative complications at baseline, and follow-up period of up to 24 months.

**Results:**

In total, 58 eyes received GATT combined with phacoemulsification surgery and 66 eyes received GATT alone. The overall qualified success rate was 86.21% for eyes with GATT combined with phacoemulsification surgery, and 83.48% for eyes with GATT only at 24 months. IOP was reduced from 26.40 ± 6.37 mmHg on 3.12 ± 0.80 medications preoperatively to 14.61 ± 2.28 mmHg on 0.27 ± 0.71 medications at 12 months and 16.08 ± 2.38 mmHg on 0.45 ± 0.96 medications at 24 months after combined surgery. Additionally, mean BCVA improved from 0.75 ± 0.43 logMAR units preoperatively to 0.22 ± 0.18 logMAR units 24 months after combined surgery. No vision-threatening complications occurred during the 24-month follow-up.

**Conclusions:**

The 24-month follow-up results of our study suggest that GATT combined with cataract surgery is a safe and effective treatment for decreasing IOP and number of medications in patients with POAG combined cataract.

## Introduction

Glaucoma and cataract are two leading causes of blindness worldwide [1–3]. As the population ages, the incidence of both blinding diseases increases. Combined surgery to treat both glaucoma and cataract has historically been of interest because of the frequent coincidence of the two conditions in older patients. The application of a combined procedure in appropriate patients will reduce surgical trauma from two separate surgeries and the high incidence of cataract development following glaucoma surgery [4].

Recently, Grover et al. [5] described a novel, minimally invasive approach termed the gonioscopy-assisted transluminal trabeculotomy (GATT) procedure, in which the trabecular meshwork (TM) is circumferentially cut off with a suture or catheter to allow the aqueous humour to pass through the collector canals and episcleral veins through the cleaved open TM. Clinical results have confirmed that GATT is a safe and effective method for reducing IOP, and it can fundamentally eliminate the postoperative complications associated with filtering blebs and reduce intraoperative bleeding [6].

Although GATT performed with cataract surgery has been reported by previous studies [5–9], the therapeutic safety and efficacy of this relatively new procedure still needs more supportive clinical evidence. Whether GATT combined with phacoemulsification and intraocular lens (IOL) implantation surgery has a more satisfactory outcome in patients with POAG combined cataract has not been well determined. Therefore, the purpose of this study was to explore the safety and effectiveness of this new minimally invasive combined surgical approach.

## Materials and methods

### Study design

This study was an observed, consecutive case series of patients with POAG who underwent microcatheter-assisted GATT or GATT combined phacoemulsification at Beijing Tongren Eye Centre between October 2019 and November 2020. This study was approved by the Institutional Ethics Committee of Beijing Tongren Hospital and conformed to the tenets of the Declaration of Helsinki. It was registered under the Chinese Clinical Trials Registry (ChiCTR1900026718). Written informed consent was obtained from all patients before surgery.

The inclusion criteria were patients with POAG (1) aged between 45 and 79 years; (2) preoperative IOP ≥ 18 mmHg with progressive deterioration of visual field (VF) defects on maximal medications, or (3) intolerance to medical therapy. Patients with other types of glaucoma, a history of ocular trauma, a history of ocular surgery other than phacoemulsification, with unidentifiable TM, corneal abnormality that did not permit visualisation of the angle or IOP measurement, and patients who were on anticoagulant therapy were excluded. POAG patients with best corrected visual acuity (BCVA) >0.3 logMAR units due to cataract were recommended to undergo GATT combined with phacoemulsification surgery. Data were collected preoperatively and at the following postoperative visits: 1, 2, 3, 7 days; 1, 3, and 6 months; thereafter, every 6 months.

Outcome measures included IOP, number of glaucoma medications, success rate, and frequency of complications. Combination glaucoma medications were enumerated according to the number of active agents in the medication. An IOP of ≥30 mmHg within 1 month of surgery was defined as an IOP spikes [10]. Microhyphema was defined as flared or layered blood ≤1 mm in the anterior chamber, and macrohyphema as layered blood >1 mm [11]. We followed the surgical success that has been previously reported [12]: (1) follow-up for at least 3 months, an IOP ≤ 18 mmHg, and a reduction of IOP by 20% or more from baseline with (qualified success) or without (complete success) glaucoma medications; or for eyes with preoperative IOP of <21 mmHg on three or four glaucoma medications but intolerant to the medications, postoperative IOP of ≤ 18 mmHg without any glaucoma medications; (2) no loss of light perception vision; and (3) no need for additional surgical procedures, which were also excluded from analysis subsequent to the intervention. The severity of glaucoma status was defined by mean deviation (MD) on Humphrey visual field 24-2 SITA-fast tests. Eyes with mild-to-moderate POAG had MD ≥− 12 dB, while eyes with severe POAG had MD <− 12 dB. Unreliable VFs defined as fixation losses >20% or false-positive responses >15% were excluded, following the manufacturer’s recommendation [13].

### Surgical procedure and postoperative care

GATT was performed as a stand-alone procedure by one of the two surgeons (NLW and HZW). A clear corneal temporal incision was created, as previously described [12], and the anterior chamber was filled with viscoelastics. A corneal paracentesis track, oriented tangentially, was then placed in the superonasal or inferonasal quadrants. A small goniotomy was created in the nasal angle and visualised with a Swan-Jacob goniolens. A microcatheter (iTrack 250 A; Ellex iScience Inc., Fremont, CA) was used for catheterisation, and circumferential trabeculotomy was then performed by pulling both ends of the microcatheter. The viscoelastic was roughly washed out, and the vcorneal wounds were hydrated to ensure watertight closure. In the combined procedures, phacoemulsification and IOL implantation were performed before GATT, and an IOL was placed in the posterior chamber of the bag. Postoperatively, topical steroid eye drops (TobraDex) were prescribed 4 times daily and then tapered over 2 to 4 weeks for all cases, with the main goal of controlling inflammation and preventing a steroid IOP response [14, 15]. If a steroid response developed within 2 weeks postoperatively or a continuous inflammatory reaction developed in anterior chamber over 4 weeks, the topical steroids were replaced by non-steroid anti-inflammatory eye drops (1% prednisolone acetate), which were administered 4 times daily until the inflammatory reaction was under control. To prevent peripheral anterior synechia, pilocarpine 2% (Bausch & Lomb, Rochester, NY, USA) was used 4 times daily for 3 months in all POAG cases with GATT or GATT combined with phaco procedures [14, 15]. When patient presented hypotony (IOP < 6 mmHg), supraciliary effusion or ciliochoroidal detachment, pilocarpine was suspended until effusion resolved spontaneously.

### Statistical analysis

Estimated sample size was calculated with G*Power (version 3.1.9.2; G*Power, Aichach, Germany). Demographic and clinical characteristics between the groups were compared using Fisher’s exact test, Chi-square test, Mann–Whitney U test, or mixed-effects models, as appropriate. The changes in IOP and the number of glaucoma medications between the groups were compared using mixed-effect models to account for the clustering of eyes within a given patient. A Kaplan–Meier survival curve was constructed to determine the cumulative probability of success. A P value of less than 0.05 (two tailed) was considered statistically significant.

## Results

### Demographics and baseline ocular characteristics

A total of 124 eyes from 124 patients were enrolled in this study (Table1). The average age was 63.83 years (range, 45–79 years), and 56 patients were male (45.16%). The mean follow-up time was 18.67 months. The eyes were divided into two groups based on the surgical procedures. Fifty-eight eyes underwent GATT combined with phacoemulsification surgery (Group 1), and the other 66 eyes underwent GATT alone (Group 2). Eyes in Group 1 had worse preoperative BCVA than the eyes in Group 2 (*P* < 0.001). The two groups did not significantly differ in terms of the mean age, sex, preoperative IOP, preoperative medications, glaucoma severity, and follow-up duration (all *P* > 0.05).

**Table 1:** Demographics and baseline ocular characteristics.

Characteristics	Total (*N* = 124)	GATT-Phaco (Group1) (*N* = 58)	GATT (Group2) (*N* = 66)	*P*-value
Age(y), mean ± SD	63.83 ± 8.63	65.02 ± 8.00	62.79 ± 9.09	0.152^a^
Range(y)	45–79	46–79	45–79	
Gender				0.472^b^
Males, *N* (%)	56 (45.16%)	24 (41.38%)	32 (48.48%)	
Females, *N* (%)	68 (54.84%)	34 (58.62%)	34 (51.52%)	
Prior glaucoma procedures, *n* (%)	12 (9.68%)	5 (8.62%)	7 (10.61%)	0.769^b^
Trabeculectomy, *n*	9	3	6	
Canaloplasty, *n*	3	2	1	
Preoperative BCVA logMAR, mean ± SD	0.62 ± 0.50	0.75 ± 0.43	0.50 ± 0.54	0.006^a^
Preoperative IOP (mmHg), mean ± SD	27.00 ± 7.33	26.40 ± 6.37	27.54 ± 8.09	0.389^d^
Preoperative antiglaucoma medications, N, mean ± SD	3.24 ± 0.73	3.12 ± 0.80	3.35 ± 0.64	0.081^d^
HVF MD values				0.971^c^
≥−12 dB, *n* (%)	63 (50.81%)	30 (51.72%)	33 (50.00%)	
<−12 dB, *n* (%)	43 (27.42%)	20 (34.48%)	23 (34.85%)	
Unreliable, *n* (%)	18 (14.52%)	8 (13.79%)	10 (15.15%)	
Follow-up duration(months), mean ± SD	18.67 ± 5.10	18.83 ± 5.01	18.53 ± 5.21	0.747^a^

*BCVA* Best-corrected visual acuity, *IOP* Intraocular pressure, *y* year, *SD* Standard deviation, *GATT-Phaco* Patients with GATT combined phacoemulsification surgery, GATT Patients with only GATT surgery.

^a^Mann–Whitney U.

^b^Fisher’s exact test.

^c^Chi-square test.

^d^Mixed-effect model.

### Surgical efficacy

**Fig. 1:**
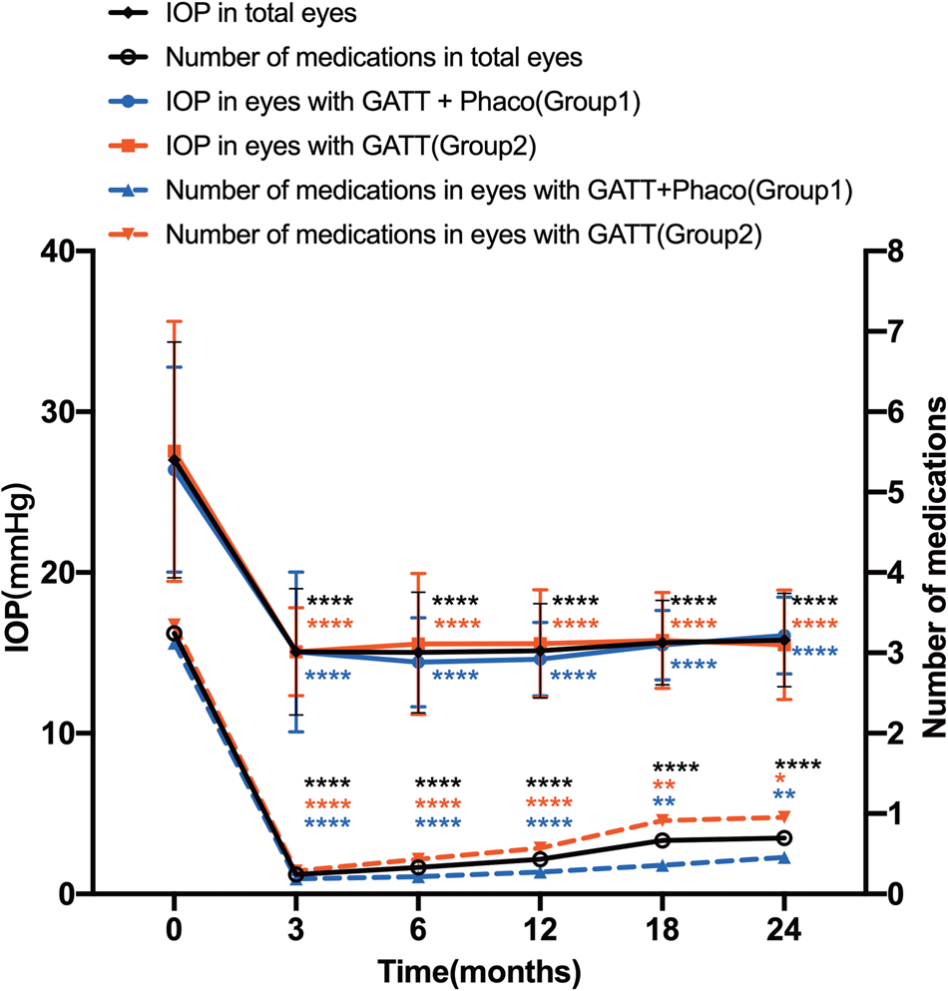
Comparison of Preoperative versus postoperative IOP and prescribed glaucoma medications for eyes that underwent GATT combined phacoemulsification surgery (Group1) and those that underwent GATT surgery only (Group2). Compared to baseline, IOP and glaucoma medication burden decreased significantly at all postoperative visits (mixed-effect model, ****P < 0.0001, **P < 0.01, *P < 0.05). The two groups did not differ significantly in terms of the mean IOP and number of prescribed glaucoma medications at each follow-up visit (mixed-effect model, *P* > 0.05).

Overall, as shown in Fig. 1, the mean IOP and number of antiglaucoma medications were significantly reduced at all postoperative visits compared to baseline (all P < 0.001). The mean IOP decreased from 27.00 ± 7.33 mmHg on 3.24 ± 0.73 medications preoperatively to 15.13 ± 2.93 mmHg on 0.43 ± 1.03 medications at 12 months and to 15.80 ± 2.90 mmHg on 0.70 ± 1.26 medications at 24 months postoperatively (Tables S1, S2). The average reduction in IOP was 43.97% and 41.50% at 12 and 24 months, respectively (Table S1). The number of glaucoma medications was reduced by an average of 2.81 and 2.54 at 12 and 24 months, respectively. The overall complete success rate (without medications) was 79.84% at 12 months and 68.42% at 24 months, and the qualified success rate (with medications) was 89.52% at 12 months and 84.81% at 24 months (Table S3).

**Fig. 2:**
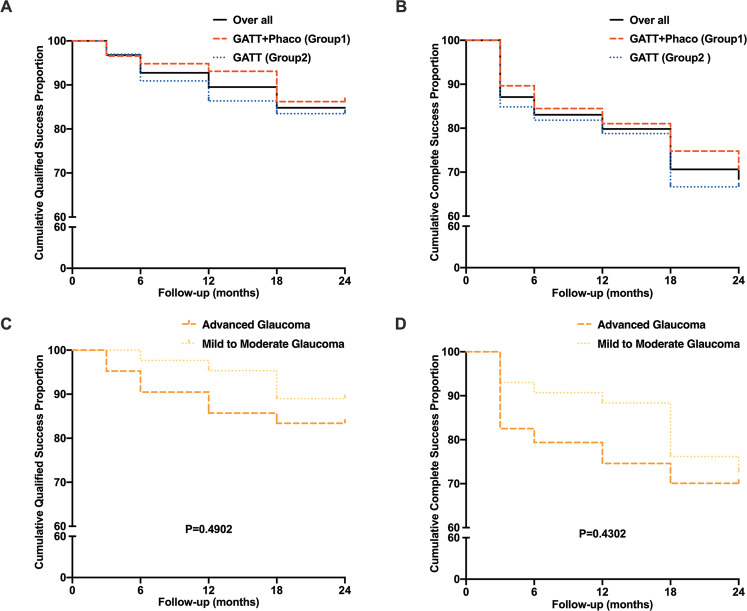
Kaplan–Meier analysis of the cumulative probabilities of surgical success. Success was defined as: an IOP of ≤18 mmHg and a reduction of IOP by 20% or more from baseline with (qualified success) or without (complete success) glaucoma medications; or for eyes with preoperative IOP of <21 mmHg on 3 or 4 glaucoma medications but intolerant to the medications, postoperative IOP of ≤18 mmHg without any glaucoma medications. Neither complete (**A**) nor qualified (**B**) success rate differed significantly between the eyes that underwent GATT combined phacoemulsification surgery or GATT only (*P* > 0.05). The difference between qualified (**C**) and complete success rate (**D**) between mild-to-moderate and advanced cases was not statistically significant. The differences between the groups were analysed by the log-rank test.

In both subgroups, the mean IOP and number of glaucoma medications decreased significantly compared to baseline (all P < 0.05; Fig. 1). In Group 1, the mean IOP decreased from 26.40 ± 6.37 mmHg on 3.12 ± 0.80 medications preoperatively to 14.61 ± 2.28 mmHg on 0.27 ± 0.71 medications postoperatively at 12 months, and to 16.08 ± 2.38 mmHg on 0.45 ± 0.96 medications at 24 months (Tables S1, S2). In the Group 2, the mean IOP decreased from 27.54 ± 8.09 mmHg on 3.35 ± 0.64 medications preoperatively to 15.57 ± 3.34 mmHg on 0.57 ± 1.22 medications postoperatively at 12 months, and 15.50 ± 3.40 mmHg on 0.95 ± 1.50 medications at 24 months (Tables S1, S2). Although the two groups did not differ significantly in terms of mean IOP or number of glaucoma medications at any follow-up visit (all *P* > 0.05; Tables S1, S2), eyes with GATT combined with phacoemulsification surgery appeared to have less medications than those with GATT surgery alone at 24 months (0.45 ± 0.96 vs. 0.95 ± 1.50, *P* = 0.557; Table S2). As shown in Fig. 2A, B, neither complete nor qualified success rates at each follow-up time point differed significantly between the two groups.

The effect of GATT on eyes with different severities of glaucoma was also considered. Although the success rate at any postoperative follow-up for advanced cases seemed to be lower than that for mild-to-moderate cases, the difference was not statistically significant (all *P* > 0.05; Fig. 2C, D). Overall, the success rates were 76.67% and 61.95% (complete success) or 86.40% and 81.82% (qualified success) for advanced cases with GATT combined with phacoemulsification surgery and with GATT alone at 24 months, respectively (all *P* > 0.05; Fig. S1A, B). The success rates were 75.00% and 68.48% (complete success) or 94.44% and 83.70% (qualified success) for mild-to-moderate cases with GATT combined with phacoemulsification surgery and with GATT alone at 24 months, respectively (all *P* > 0.05; Fig. S1C, D).

### Visual acuity

**Fig. 3:**
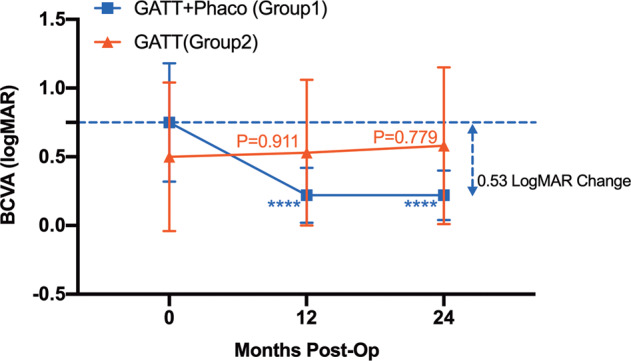
Preoperative versus postoperative BCVA in eyes that underwent GATT combined phacoemulsification surgery (Group 1) and those that underwent GATT surgery only (Group 2). Compared to baseline, BCVA improved significantly at last postoperative visits in eyes that underwent GATT combined phacoemulsification surgery (Mann–Whitney U test, ****P < 0.0001). The eyes that underwent GATT surgery only did not differ significantly between preoperative BCVA and postoperative BCVA (Mann–Whitney U test).

As shown in Fig. 3, the mean BCVA was increased by 0.03 logMAR (*P* = 0.911) and 0.05 logMAR (*P* = 0.779) at 12 months and 24 months after GATT surgeries. Concurrently, a mean improvement in BCVA of 0.53 logMAR was observed at 12 months and 24 months after GATT combined with phacoemulsification surgery (*P* < 0.0001; Table S4). Moreover, the BCVA was reduced to <0.3 logMAR in 35 eyes (60.34%) at 12 months and 12 eyes (54.55%) at 24 months among the 58 eyes with preoperative BCVA ≥ 0.3 logMAR (Table S4).

### Complications and reoperation

Surgical and postoperative complications are shown in Table 2, No patient experienced any vision-threatening complications during the entire follow-up period. Microhyphema occurred in 46 (39.5%) eyes on the first postoperative day, and macrohyphema occurred in another 50 eyes (40.3%). The incidence of hyphema did not differ between eyes with GATT combined with phacoemulsification surgery and eyes with GATT surgery (*P* = 0.769). The hyphema resolved in the vast majority of eyes in the first 2 weeks postoperatively. Ten (17.2%) eyes with GATT combined with phacoemulsification surgery experienced an IOP spike, which was significantly less than 36 (54.5%) eyes with GATT surgery only. Mostly, IOP spikes are controlled by glaucoma medications or by releasing aqueous humour from the paracentesis site created during surgery. Only 2 (3.0%) eyes with GATT surgery alone underwent anterior chamber washout because of massive hyphema and IOP spike. The proportion of eyes with combined surgery that demonstrated supraciliary effusion (*n* = 15 [25.9%]) was slightly less than that with GATT surgery alone (*n* = 26 [39.4%]). Among all the patients that experienced supraciliary effusion, only 3 cases (2 with GATT surgery only) were detected an obvious cyclodialysis cleft, which indicates the occurrence of ciliochoroidal detachment. Supraciliary effusion and ciliochoroidal detachment resolved within the first month after surgery in all cases. In total, three eyes underwent secondary surgery for IOP control (two goniosynechialysis and one trabeculectomy), including one with combined surgery and two with GATT surgery alone.

**Table 2:** Surgical and postsurgical ocular-related complications.

Complication	Total *n*(%)	GATT-Phaco *n*(%)	GATT *n*(%)	*P*-value
Hyphema				0.769^a^
Microhyphema	49 (39.5)	21 (36.2)	28 (42.2)	
Macrohyphema	50 (40.3)	25 (43.1)	25 (37.9)	
Not observed	25 (20.2)	12 (20.7)	13 (19.7)	
IOP spike	46 (37.1)	10 (17.2)	36 (54.5)	<0.0001^b^
Supraciliary effusion	41 (33.1)	15 (25.9)	26 (39.4)	0.128^b^
Descemet membrane detachment	0	0	0	
Iris prolapse	0	0	0	
Corneal edema	0	0	0	

^a^Chi-square test.

^b^Fisher’s exact test.

## Discussion

Micro-invasive glaucoma surgery (MIGS) has exploded over the last eight years [16]. GATT, an important member of the MIGS family, is characterised as a conjunctival-sparing surgical procedure that is safer and less invasive than conventional filtration surgery [6, 17–19].

A previous study demonstrated that GATT alone was safe and effective for 68% to 90% of eyes in a variety of clinical settings [6, 7], including eyes with prior incisional glaucoma surgery [16]. Our results with ab interno trabeculotomy using a microcatheter showed comparable success rates of 89.52% and 84.80% (qualified success) or 79.84% and 68.42% (complete success) at 12 and 24 months, respectively. MIGS is usually recommended for patients with early or moderate glaucoma. Grover et al. [20] reported that eyes with advanced POAG (MD of −15.0 or worse) had more than 80% failure at 6 months after GATT. However, our results showed that GATT was effective for at least 61.95% of advanced POAG, which was consistent with previous studies by our teammates Wang YW et al. [12] and Aktas et al. [21].

Recently, combined non-penetrating glaucoma surgery and cataract surgery have been extensively studied [6–9, 22]. A clinical study of canaloplasty combined with phacoemulsification cataract surgery found a significantly greater IOP reduction in combined surgical cases than in cases of canaloplasty alone [23, 24]. In several studies, the GATT combined with phacoemulsification cataract surgery has been reported to be safe and effective in treating POAG with cataract cases [6, 8, 9], which has an average postoperative IOP of 15.2 ± 4.3 mmHg (on 0.7 medications) at 12 months and 14.1 ± 3.2 mmHg (on 1.0 medications) at 24 months after GATT combined surgery. Different from previous studies, we found that the number of glaucoma medications were relatively less in cases with GATT combined with phacoemulsification surgery (0.27 ± 0.71 at 12 months and 0.45 ± 0.96 at 24 months) compared to cases with GATT alone (0.57 ± 1.22 at 12 months and 0.95 ± 1.50 at 24 months). It has been established that cataract surgery alone produces a postoperative reduction in IOP that ranges from approximately 1 to 5 mmHg [25, 26]. However, the cases with GATT combined with phacoemulsification surgery and those with GATT surgery alone finally reached the same level of IOP in both previous studies [6, 9] and our study. The mechanism of IOP reduction after cataract surgery has been postulated to be associated with increased outflow facilities due to tensioning of the TM [26, 27]. GATT surgery removes the resistance formed by the TM by opening the TM, which alters the original anatomy [28]. This may explain why eyes with combined surgery did not reach a lower level of IOP than those with GATT alone.

The success percentiles (qualified success) were reported not to be significantly different between GATT (87.5%) and GATT with phacoemulsification surgery (83.8%) in previous studies [9]. In our study, the success rates of eyes with combined procedures were consistent with those of previous studies and did not differ from those of eyes with GATT alone.

A previous study [6, 7, 9] found that long-term visual acuity did not worsen significantly after GATT surgery, except for patients with advanced glaucoma and uncontrolled IOP, which had a high risk of vision loss. In our study, only two advanced cases receiving GATT surgery lost three lines of acuity, and no cases had complete vision loss. For eyes with coexisting cataracts and POAG, cataract extraction with intraocular lens implantation is essential for visual improvement. Based on our observations, cases with combined surgery improved five lines of corrected visual acuity compared with preoperative BCVA. Postoperative complications have been reported to have a marked effect on early visual acuity after combined surgery but do not influence long-term restoration of visual acuity [6]. This point of view is also indirectly confirmed in our research.

In our study, the rate of ciliochoroidal detachments was 1.7% and 3.0% for combined surgeries and GATT, respectively. Moreover, the incidence of supraciliary effusion was 25.9% and 39.4% for combined surgeries and GATT, respectively. The individual incidences of supraciliary effusion and ciliochoroidal detachment after GATT surgeries reported in another study by our team were 57% and 3%, respectively [15], which is consistent with this study. Other previous studies have also shown that supraciliary effusion is a common postoperative complication after 360-degree trabeculotomy. Sato et al. [29] reported that 47.7% of the patients developed supraciliary effusion after 360-degree trabeculotomy. Akagi et al. [30] demonstrated the presence of supraciliary effusion in 42% patients who underwent ab interno trabeculotomy. Altogether, the incidence of supraciliary effusion in this study was consistent with other previously reported studies. Compared to supraciliary effusion, ciliochoroidal detachment complication is rare after GATT surgeries. Previous studies conducted by Gover et al. [5, 6, 20] did not report the occurrence of ciliochoroidal detachment. The effect of pilocarpine on the development of supraciliary effusion or ciliochoroidal detachment cannot be excluded in our study. Although, we observed that supraciliary effusion and choroidal detachment usually happened on the first day after operation; supraciliary effusions may occur mainly due to traction on the ciliary muscle fibres when the catheter is pulled to create the trabeculotomy incision during the operation. As for the three cases of ciliochoroidal detachment, they were all detected in the nasal quadrant, which may have probably been caused by direct trauma from the blade when a nasal goniotomy incision is made. Notably, in present study, supraciliary effusion and ciliochoroidal detachment resolved within the first month after surgery in all cases and did not result in a significant worsening of final visual acuity.

Our study had some limitations such as the relatively small sample size, the limited follow-up time, and the non-randomized controlled study design, which may have weakened our research. However, our study may contribute to the evidence on the IOP-lowering efficacy and safety of GATT combined phacoemulsification as a viable option for patients with POAG and cataract. To further understanding on this topic and to assess the advantages of this procedure, long-term investigations using randomized clinical trial designs and larger sample sizes are needed.

In conclusion, GATT is a MIGS that attempts to disarm resistance from the TM and restore a more natural outflow of aqueous fluid within the eye. Interim analysis of this observational, consecutive case series clinical study indicated that the surgical procedure in combination with phacoemulsification and IOL implantation effectively lowers IOP with few long-term complications and with continued control of IOP in patients followed up to 24 months. The procedure was safe and vision-improving for the majority of cases with POAG combined with cataract.

Supplemental material is available on Eye’s website.

## Summary

### What was known before

- Clinical results have confirmed that GATT is a safe and effective method for reducing IOP, and it can fundamentally eliminate the postoperative complications associated with filtering blebs and reduce intraoperative bleeding.

### What this study adds

- The 24-month follow-up results of our study suggest that GATT combined with cataract surgery is a safe and effective treatment for decreasing IOP and number of medications in patients with POAG combined cataract.

## Supplementary information

Supplement Table.1. Preoperative and postoperative IOP levels

Supplement Table.2. Preoperative and postoperative number of anti-glaucoma medications

Supplement Table.3. Kaplan–Meier analysis of the cumulative probabilities of surgical success.

Supplement Table.4. Preoperative versus postoperative BCVA in eyes with GATT combined phacoemulsification surgery (Group1) and those with GATT surgery only (Group2).

Fig.S1. Kaplan–Meier analysis of the cumulative probabilities of surgical success in mild-to-moderate and severe POAG.

## Data Availability

All data generated or analysed during this study are included in this published article [and its supplementary information files].
