# Supplementary material for: Gonioscopy-assisted Transluminal Trabeculotomy (GATT) combined phacoemulsification surgery: Outcomes at a 2-year follow-up
Source: Eye (Lond). 2022 May 24;37(6):1258–63. doi: 10.1038/s41433-022-02087-2 (PMC10102214; doi:10.1038/s41433-022-02087-2)
Supplement: Supplementary file 1 — Supplement Table.1. Preoperative and postoperative IOP levels [file 41433_2022_2087_MOESM1_ESM.docx]

**Supplement Table.1. Preoperative and postoperative IOP levels**

| **IOP** | **Total** | | **GATT-Phaco (Group 1)** | | **GATT (Group 2)** | | **P value^a^** |
| --- | --- | --- | --- | --- | --- | --- | --- |
|  | **Mean±SD** | **Decreased (%)**  **Compared to preoperation** | **Mean±SD** | **Decreased (%)**  **Compared to preoperation** | **Mean±SD** | **Decreased (%)**  **Compared to preoperation** |  |
| Preoperative | 27.00 ± 7.33 |  | 26.40 ± 6.37 |  | 27.54 ± 8.09 |  | 0.6505 |
| Postoperative |  |  |  |  |  |  |  |
| 3 months | 15.06 ± 3.92 | 44.22 | 15.06 ± 4.97 | 42.96 | 15.07 ± 2.73 | 45.28 | >0.9999 |
| 6 months | 15.03 ± 3.75 | 44.35 | 14.42 ± 2.77 | 45.39 | 15.56 ± 4.38 | 43.51 | 0.6656 |
| 12 months | 15.13 ± 2.93 | 43.97 | 14.61 ± 2.28 | 44.67 | 15.57 ± 3.34 | 43.45 | 0.8106 |
| 18 months | 15.64 ± 2.63 | 42.08 | 15.48 ± 2.15 | 41.36 | 15.77 ± 2.98 | 42.73 | >0.9999 |
| 24 months | 15.80 ± 2.90 | 41.50 | 16.08 ± 2.38 | 39.09 | 15.50 ± 3.40 | 43.70 | 0.9989 |

a Two-way ANOVA analysis (Mixed-effect model) between Group 1 and Group 2 at each follow-up time points
