# Supplementary material for: Gonioscopy-assisted Transluminal Trabeculotomy (GATT) combined phacoemulsification surgery: Outcomes at a 2-year follow-up
Source: Eye (Lond). 2022 May 24;37(6):1258–63. doi: 10.1038/s41433-022-02087-2 (PMC10102214; doi:10.1038/s41433-022-02087-2)
Supplement: Supplementary file 2 — Supplement Table.2. Preoperative and postoperative number of anti-glaucoma medications [file 41433_2022_2087_MOESM2_ESM.docx]

**Supplement Table.2. Preoperative and postoperative number of anti-glaucoma medications**

| **Glaucoma medications** | **Total** | | **GATT-Phaco (Group 1)** | | **GATT (Group 2)** | | **P value^a^** |
| --- | --- | --- | --- | --- | --- | --- | --- |
|  | **Mean±SD** | **Decreased compared to preoperative** | **Mean±SD** | **Decreased compared to preoperative** | **Mean±SD** | **Decreased compared to preoperative** |  |
| Preoperative | 3.24 ± 0.73 |  | 3.12 ± 0.80 |  | 3.35 ± 0.64 |  | 0.657 |
| Postoperative |  |  |  |  |  |  |  |
| 3 months | 0.24 ± 0.71 | 3.00 | 0.19 ± 0.66 | 2.93 | 0.29 ± 0.76 | 3.06 | 0.991 |
| 6 months | 0.33 ± 0.84 | 2.91 | 0.21 ± 0.56 | 2.91 | 0.43 ± 1.02 | 2.92 | 0.720 |
| 12 months | 0.43 ± 1.03 | 2.81 | 0.27 ± 0.71 | 2.85 | 0.57 ± 1.22 | 2.78 | 0.373 |
| 18 months | 0.67 ± 1.19 | 2.58 | 0.36 ± 0.87 | 2.76 | 0.91 ± 1.36 | 2.43 | 0.091 |
| 24 months | 0.70 ± 1.26 | 2.54 | 0.45 ± 0.96 | 2.67 | 0.95 ± 1.50 | 2.40 | 0.363 |

a Two-way ANOVA analysis (Mixed-effect model) between Group 1 and Group 2 at each follow-up time points
