# Supplementary material for: Gonioscopy-assisted Transluminal Trabeculotomy (GATT) combined phacoemulsification surgery: Outcomes at a 2-year follow-up
Source: Eye (Lond). 2022 May 24;37(6):1258–63. doi: 10.1038/s41433-022-02087-2 (PMC10102214; doi:10.1038/s41433-022-02087-2)
Supplement: Supplementary file 3 — Supplement Table.3. Kaplan–Meier analysis of the cumulative probabilities of surgical success. [file 41433_2022_2087_MOESM3_ESM.docx]

**Supplement Table.3. Kaplan–Meier analysis of the cumulative probabilities of surgical success.**

| **Success proportion (%)** | **Total** | | **GATT-Phaco (Group 1)** | | **GATT (Group 2)** | |
| --- | --- | --- | --- | --- | --- | --- |
|  | **Complete** | **Qualified** | **Complete** | **Qualified** | **Complete** | **Qualified** |
| 3 months | 87.10 | 96.77 | 89.66 | 96.55 | 84.85 | 96.97 |
| 6 months | 83.06 | 92.74 | 84.48 | 94.83 | 81.82 | 90.91 |
| 12 months | 79.84 | 89.52 | 81.03 | 93.10 | 78.79 | 86.37 |
| 18 months | 70.63 | 84.80 | 74.80 | 86.21 | 66.67 | 83.48 |
| 24 months | 68.42 | 84.80 | 70.65 | 86.21 | 66.67 | 83.48 |
