# Supplementary material for: Gonioscopy-assisted Transluminal Trabeculotomy (GATT) combined phacoemulsification surgery: Outcomes at a 2-year follow-up
Source: Eye (Lond). 2022 May 24;37(6):1258–63. doi: 10.1038/s41433-022-02087-2 (PMC10102214; doi:10.1038/s41433-022-02087-2)
Supplement: Supplementary file 4 — Supplement Table.4. Preoperative versus postoperative BCVA in eyes with GATT combined phacoemulsification surgery (Group1) and those with GATT surgery only (Group2). [file 41433_2022_2087_MOESM4_ESM.docx]

**Supplement Table.4. Preoperative versus postoperative BCVA in eyes with GATT combined phacoemulsification surgery (Group1) and those with GATT surgery only (Group2).**

|  | **Mean BCVA**  **(LogMAR)** ± **SD** | | | **Eyes with BCVA**  **<0.3 LogMAR, n (%)** | |
| --- | --- | --- | --- | --- | --- |
|  | **GATT-Phaco** | **GATT** |  | **GATT-Phaco** | **GATT** |
| Preoperative | 0.75 ± 0.43 | 0.50 ± 0.54 | 0/58 (0.00) | | 33/66(50.00) |
| Postoperative |  |  |  | |  |
| 12 months | 0.22 ± 0.20 | 0.53 ± 0.53 | 35/58 (60.34) | | 31/66(46.97) |
| 24 months | 0.22 ± 0.18 | 0.58 ± 0.57 | 12/22 (54.55) | | 10/21(47.62) |
