# Supplementary figures and images for: Gonioscopy-assisted Transluminal Trabeculotomy (GATT) combined phacoemulsification surgery: Outcomes at a 2-year follow-up
Source: Eye (Lond). 2022 May 24;37(6):1258–63. doi: 10.1038/s41433-022-02087-2 (PMC10102214; doi:10.1038/s41433-022-02087-2)

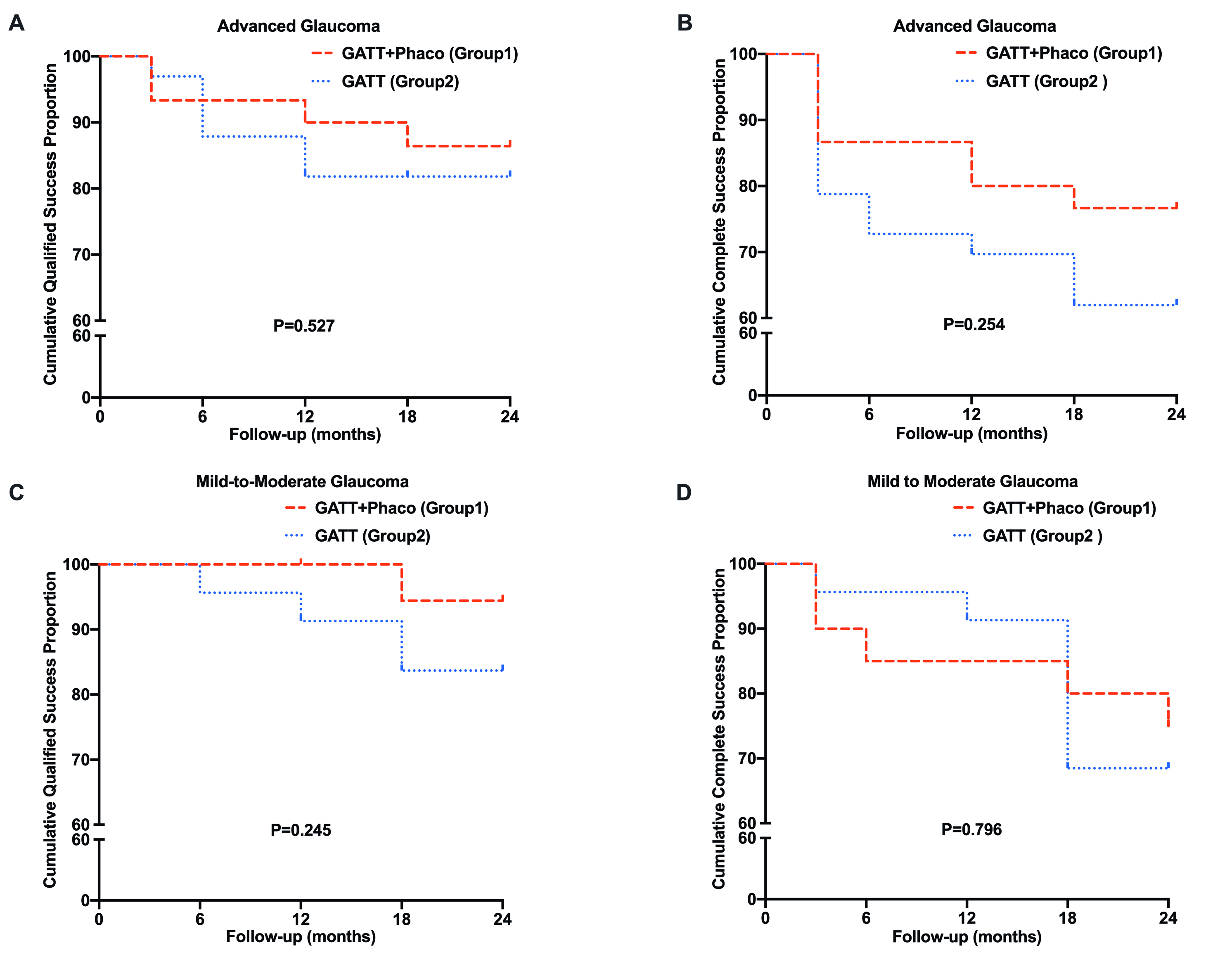

Supplement: Supplementary file 5 — Fig.S1. Kaplan–Meier analysis of the cumulative probabilities of surgical success in mild-to-moderate and severe POAG. [file 41433_2022_2087_MOESM5_ESM.tif]
